# Melittin suppresses tumor progression by regulating tumor-associated macrophages in a Lewis lung carcinoma mouse model

**DOI:** 10.18632/oncotarget.18627

**Published:** 2017-06-27

**Authors:** Chanju Lee, Sung-Joo S. Bae, Hwansoo Joo, Hyunsu Bae

**Affiliations:** ^1^ Department of Physiology, College of Korean Medicine, Kyung Hee University, Dongdaemoon-Gu, Seoul 02447, Republic of Korea; ^2^ Department of Biology, University of California Riverside, Riverside, CA 92521, USA

**Keywords:** melittin, tumor-associated macrophages (TAM), CD206

## Abstract

Tumor-associated macrophages (TAM) are a major component of tumor stroma. It has been reported that TAMs have M2-like phenotype and facilitate tumor progression by promoting angiogenesis and immunosuppression. Melittin, a major polypeptide of bee venom, has been widely studied as an anti-cancer drug due to its cytotoxicity to malignant cells. However, very little is known regarding the effect of melittin on immune cells in the tumor microenvironment. This study focuses on the effect of melittin on TAMs in a Lewis lung carcinoma mouse model. Melittin inhibited the rapid tumor growth compared to the control *in vivo*. Melittin increased the M1/M2 ratio of TAMs by selectively reducing the number of CD206^+^ M2-like TAMs while not altering the population of CD86^+^ M1-like TAMs. Melittin also preferentially binds to M2 macrophages, and this binding was not associated with phagocytosis. Gene and protein expression of vascular endothelial growth factor (Vegf) and mannose receptor C type 1 (Mrc1/CD206) was reduced in M2-like bone marrow-derived macrophages by melittin treatment, but there was no significant change in the gene level of Vegf and FMS-like tyrosine kinase 1 (Flt1/VEGFR1) in tumor cells *in vitro*. Additionally, the levels of VEGF and CD31, markers of angiogenesis, were significantly decreased by melittin treatment in tumor tissues. This study revealed a novel role for melittin in tumor treatment and suggested that melittin could be a promising therapeutic agent for targeting M2-like TAMs.

## INTRODUCTION

The tumor microenvironment which contributes to proliferation and survival of malignant cells, angiogenesis, metastasis, abnormal adaptive immunity and reduced response to hormones and chemotherapeutic agents is largely being considered as a therapeutic target [1, 2]. A number of studies have demonstrated that tumor associated macrophages (TAM) are major subsets of tumor microenvironment and a key regulator of angiogenesis which is an essential process for tumor progression by supplying oxygen and nutrients into hypoxic tumor area [3–5]. The role of TAMs in the tumor microenvironment still remains controversial. TAMs are categorized as two different phenotypes: tumor-suppressive M1 or tumor-supportive M2 macrophages. M1-like TAMs have powerful ability to present antigens and generally exhibit CD86 and TNF-α. In contrast, M2-like TAMs have weak antigen presenting function and high phagocytosis capacity. They are known to be immunosuppressive, pro-tumorigenic, pro-angiogenic by releasing various extracellular matrix components, angiogenic and chemotactic factors. M2-like TAMs are distinguished with M1-like TAMs by expressing some markers like CD163, CD204, CD206, IL-10 [6–9]. In most tumors such as breast, ovarian, prostate, lung carcinoma, and cutaneous melanoma, the tumor microenvironment includes a number of factors, such as CSF-1, VEGF, CCL2, IL-4, IL-13, TGF-β, and IL-10, which can recruit monocytes and lead to differentiation for M2-like phenotype [10–12].

Previous research shows that depletion of macrophages by encapsulated clodronate could reduce angiogenesis in tumor tissue [13]. In addition, blocking the infiltration of macrophages via CSF-1R and CCR2 antibodies could reduce the tumor-initiating properties and increase the activity of cytotoxic T lymphocytes [14]. Targeting M2-like TAMs is therefore a potential therapeutic strategy to combat tumor progression.

Melittin is a major component of honey bee (*Apis mellifera* L.) venom and an amphiphilic peptide that has 26 amino acid residues [15]. Melittin has membrane-perturbing effects, such as pore formation, fusion and vesiculation [16–18]. Melittin has been used in tumor bearing mice research because of its cytotoxicity to tumor cells and capacity to inhibit cell growth or induce apoptosis and necrosis [19–21]. However, it is a very nonspecific cytolytic peptide, and can cause off-target effects attacking all lipid membranes and disrupting the membranes of normal cells [22–24]. On the other hand, there is a report that a low dose of melittin is able to prevent pore formation [25]. Melittin is reported to have the capacity to neutralize the inflammatory activity of macrophages by regulating intracellular factors, such as p50 and IκB kinase-α [26]. It also has been shown that melittin interacts with LPS and inhibits the LPS-induced activation of macrophages and inflammatory cytokine production [27, 28]. However, the role of melittin in the regulation of macrophage activity in the tumor microenvironment is still unknown. In this study, we investigate the novel role of melittin on TAMs and show that melittin reduced the CD206^+^ TAMs, but not CD86^+^ TAMs through regulating the gene expression of TAMs in a Lewis lung carcinoma (LLC) mouse model.

## RESULTS

### Melittin suppresses tumor growth and improves survival *in vivo*

**Figure 1:**
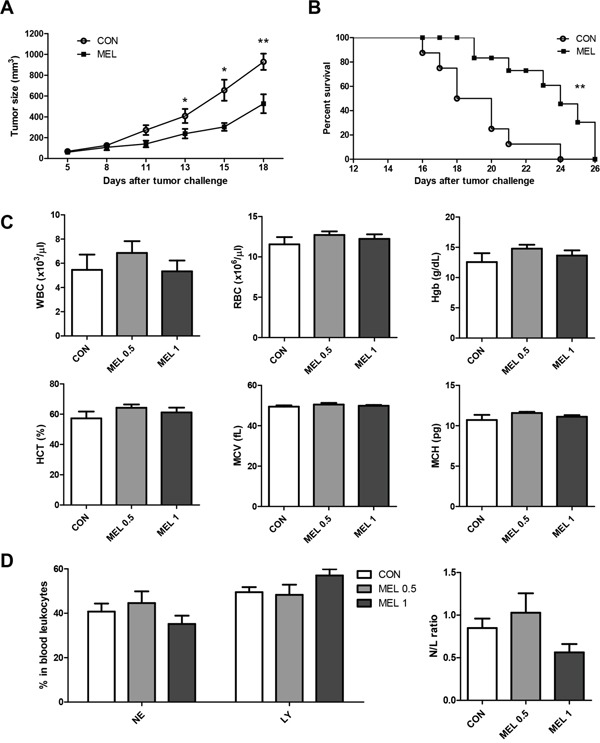
Antitumor effect of melittin *in vivo* **(A)** Tumor sizes of control (CON) and melittin (MEL) groups were measured and calculated. Tumor-bearing mice were administered the melittin peptide (0.5 mg/kg) every 3 days (an overall total of five times) starting on day 5 after tumor inoculation. N=5 animals per group. **(B)** Mantel-Cox survival curve. N=8 animals per group. **(C)** Hematological profiling. Blood was collected and parameters for myeloid function were analyzed. **(D)** Percentage of neutrophils and lymphocytes in blood leukocytes from tumor bearing mice (left). Neutrophil/lymphocyte ratio (N/L ratio) was used to verify whether acute cytotoxicity occurred as a result of melittin treatment (right). All of the data are presented as the mean ± SEM. *P < 0.05, **P < 0.01.

To test whether melittin can suppress Lewis lung carcinoma (LLC) tumor growth *in vivo*, we subcutaneously injected tumor cells into C57BL/6 mice and treated them with i.p. injections of 0.5 mg/kg of melittin peptide or PBS every 3 days. The tumor growth rate of the control group was rapid, whereas the growth of the melittin treated group was delayed. Thirteen days after the challenge, tumor growth of the melittin treated group was significantly reduced by approximately 40% compared to the control group and suppressed by approximately 45% at day 15 (Figure 1A). In addition, the melittin treatment significantly prolonged the survival time in a Mantel-Cox analysis when compared with control PBS treatment (Figure 1B). The body weights of the mice were recorded from day 0 to 18 every 2-3 days. There was no body weight loss (control: 22.44 ± 0.37 at day 0 and 23.6 ± 0.30 at day 18; melittin: 22.84 ± 0.36 at day 0 and 24.72 ± 0.45 at day 18) in both the control and melittin treated group (data not shown). To confirm the side effects of melittin on myeloid function, hematological profiling was performed. Blood was collected from the retro-orbital plexus under anesthesia. Treatment with 0.5 mg/kg or 1 mg/kg of melittin did not result in significant changes in hematological parameters, including the level of white blood cells (WBC), red blood cells (RBC), hemoglobin (Hgb), hematocrit (HCT), mean corpuscular volume (MCV), and mean corpuscular hemoglobin (MCH) (Figure 1C). In addition, the percentage of neutrophils and lymphocytes and the neutrophil-lymphocyte ratio (N/L ratio) were not significantly changed, suggesting that there was no acute cytotoxic injury (Figure 1D) [29].

### Melittin treatment does not directly cause cell cycle arrest of tumor cell

**Figure 2:**
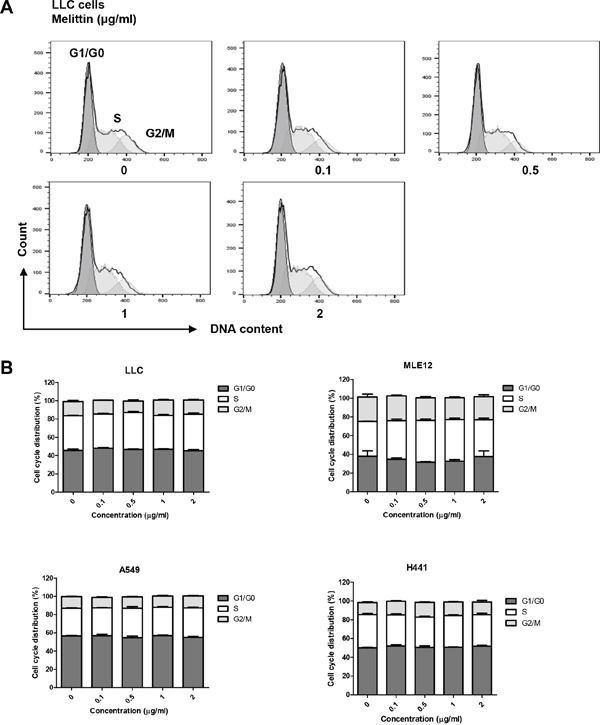
Effect of melittin on cell cycle of tumor cells *in vitro* **(A-B)** The cell cycle was detected by PI staining and plotted as a histogram after gating on single cells. Representative figures of three replicates for the cell cycle are shown. The peaks correspond to the G1/G0, S, and G2/M phases. The results are given as the mean ± SEM. *P < 0.05, **P < 0.01, ***P < 0.001.

PI-stained lung tumor cells were detected by flow cytometry to verify the effect of melittin on the cell cycle of tumor cells. There was no change in the percentage of each phase (Figure 2). These results showed that 0.1 - 2 μg/ml of melittin did not affect cell cycle of tumor cells *in vitro*.

### Treatment with melittin decreases the number of macrophages in the tumor microenvironment *in vivo*

**Figure 3:**
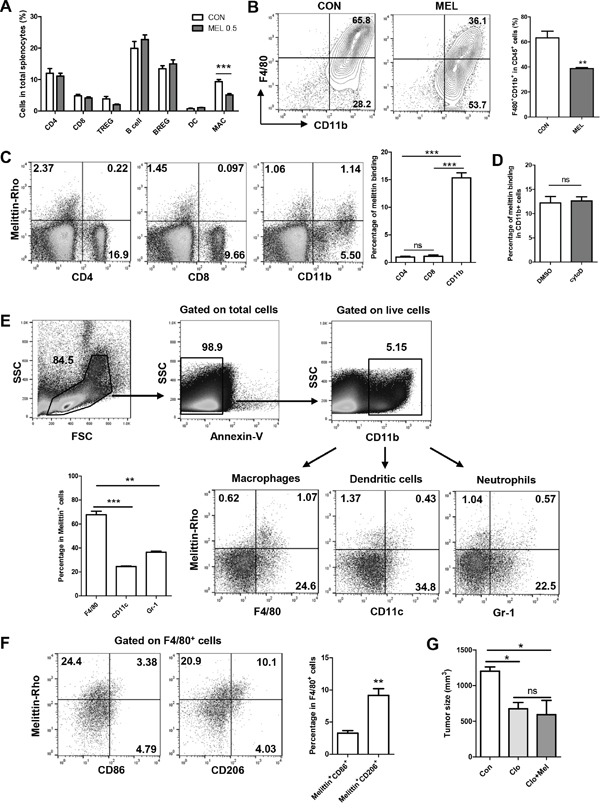
Selective reduction of the number of TAMs by melittin treatment **(A)** Splenocytes from tumor bearing mice were stained for detecting CD4 T cells (CD3^+^CD4^+^CD8^-^), CD8 T cells (CD3^+^CD8^+^CD4^-^), regulatory T cells (TREG; CD4^+^CD25^+^Foxp3^+^), B cells (B220^+^CD19^+^CD25^-^), regulatory B cells (BREG; B220^+^CD19^+^CD25^+^), dendritic cells (DC; CD45^+^CD11b^+^CD11c^+^), and macrophages (MAC; CD45^+^F4/80^+^). Ratios of each immune cell in splenocytes were determined by flow cytometric analysis. **(B)** TAMs from tumor tissue were marked as CD11b^+^F4/80^+^ and presented as a contoured line (left) after gating on CD45^+^ cells from total live gated cells. The percentages of CD11b^+^F4/80^+^ cells in CD45^+^ cells are shown as a bar graph (right). **(C)** Binding of melittin to CD4^+^, CD8^+^ and CD11b^+^ cells in a mixed population of splenocytes was detected. **(D)** Percentages of melittin^+^CD11b^+^ cells in total CD11b^+^ cells treated with either DMSO or cytochalasin D (Cyto D) were measured to verify whether the co-staining was associated with phagocytosis. **(E)** Melittin binding subsets of CD11b^+^ cells were identified as F4/80^+^ macrophages, CD11c^+^ dendritic cells, and Gr-1^+^ neutrophils following a gating strategy. **(F)** Subpopulations of melittin-bound macrophages were identified as CD86^+^ (M1) and CD206^+^ (M2). All plots are representative of three replicates. **(G)** Tumor size was monitored after macrophage depletion. Clodronate liposome (Clo) or vehicle liposome (Con) was injected by i.p. 3 days before tumor inoculation, followed by treatment every 4 days. 0.5mg/kg of melittin was co-treated with Clo (Clo+Mel). N=3-4 animals per group. All of the values are shown as the mean ± SEM. **P <0.01, ***P < 0.001.

We examined if there was any change in the immune cells with melittin treatment. Screening of immune cells was performed for CD4^+^ T cells, CD8^+^ T cells, B cells, dendritic cells and macrophages in splenocytes from tumor bearing mice by flow cytometry. Melittin markedly reduced only the percentage of CD45^+^F480^+^ macrophages (9.05 ± 0.80 for control vs. 5.10 ± 0.42 for melittin). The population of regulatory T cells decreased slightly, but this change was not significant. There were no differences between the control and melittin-treated group in the percentages of the other immune cells in total splenocytes (Figure 3A). Next, the effect of melittin on TAMs in the tumor was investigated. With melittin treatment, the percentage of CD11b^+^F4/80^+^ macrophages in CD45^+^ tumor infiltrating leukocytes was significantly decreased (63.25 ± 5.34 for control vs. 38.70 ± 0.79 for melittin) (Figure 3B). To ascertain whether the TAM-regulating role of melittin was involved in tumor growth inhibition, macrophage was depleted by clodronate liposome. As expected, macrophage depletion by clodronate significantly reduced the tumor growth compared with control. Melittin did not further suppress tumor growth upon co-treatment with clodronate, suggesting that the tumor inhibiting effects of melittin is closely associated with TAM (Figure 3G).

### Melittin peptide binds preferentially to M2 macrophages within mixed population of cells *in vitro*

Binding tests were performed to determine whether melittin was able to bind selectively to CD11b^+^ macrophages. Splenocytes were incubated with rhodamine-conjugated melittin peptide and stained with CD4, CD8, and CD11b antibodies. Melittin binding was exhibited in approximately 16% in the CD11b^+^ cells, whereas binding was only observed in 1% in the CD4^+^ or CD8^+^ cells (Figure 3C). To verify whether the detection of peptide in CD11b^+^ cells is related to phagocytosis, splenocytes were pretreated with 10 nM cytochalasin D (Cyto D), an inhibitors of actin polymerization, before melittin treatment to inhibit phagocytosis. There was no difference between the DMSO control and Cyto D group on the amount of melittin^+^CD11b^+^ double positive cells in the total CD11b^+^ cell population (Figure 3D). These results show that melittin has an affinity for CD11b^+^ cells, and this affinity was not associated with phagocytosis.

Next, we investigated melittin binding subset of CD11b^+^ cells by staining F4/80, CD11c, and Gr-1 for macrophages, DCs, and neutrophils respectively. Melittin showed preferential binding to macrophages (67.70 ± 2.96 in the Annexin-V-CD11b^+^melittin^+^ cells) whereas low binding was exhibited to DCs (24.44 ± 0.56) and neutrophils (36.36 ± 0.95) (Figure 3E). We further confirmed that melittin preferentially bound to M2 macrophages. For phenotypic characterization of melittin-bound macrophage, M1 type was marked as CD86 and M2 type was marked as CD206. The percentage of melittin and CD206 double positive populations in F4/80^+^ cells was significantly higher (9.15 ± 1.05) than the percentage of melittin^+^CD86^+^ populations (3.27 ± 0.39) (Figure 3F).

### Melittin increases the M1/M2 ratio by reducing the number of M2-like TAMs specifically

**Figure 4:**
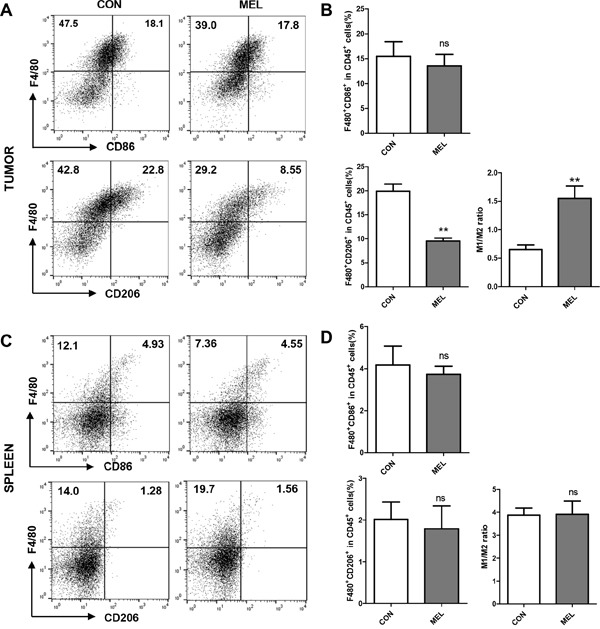
Improvement of M1/M2 ratio by decrease of M2-like CD206^+^ TAM in tumor *in vivo* **(A-B)** M1-like macrophages that infiltrated tumor cells were marked as F4/80^+^CD86^+^ (upper panel), and M2-like TAMs were marked as F4/80^+^CD206^+^ (lower panel). **(C-D)** F4/80^+^CD86^+^ and F4/80^+^CD206^+^ macrophages in splenocytes are shown. The M1/M2 ratios were calculated based on the dot plots of CD86^+^ (M1) and CD206^+^ (M2) cells in F4/80^+^ macrophages. All plots were gated on CD45^+^ cells from total live gated cells. The values are presented as the mean ± SEM; ** P <0.01.

Although both M1-like and M2-like macrophages are present in tumor tissue, pro-angiogenic TAMs are considered the M2-like phenotype [10, 11]. Several studies have shown that a high M1/M2 ratio of macrophages improved the survival rate in some human cancer models [30–32]. Although there was no increase in the F4/80^+^CD86^+^ population in CD45^+^ cells, the percentage of F4/80^+^CD206^+^ cells in CD45^+^ cells was significantly decreased from 19.90 ± 1.49 for the control group to 9.53 ± 0.63 for the melittin treated group (Figure 4A and 4B). Interestingly, the levels of M1 or M2-like macrophages in splenocytes were unchanged by melittin treatment (Figure 4C and 4D). As a result, the M1/M2 ratio in the tumor tissues was markedly increased by melittin treatment (M1/M2 = 0.65 for control vs. M1/M2 = 1.55 for melittin injection) (Figure 4B, right panel), but the ratio in the spleen (approximately 3.9) remained unchanged (Figure 4D, right panel). This observation indicated that melittin is capable of considerably increasing the M1/M2 ratio as a therapeutic indicator by specifically reducing the M2-like TAMs in tumor tissue, while not affecting the M1-like or spleen resident macrophage populations *in vivo*.

### Melittin treatment reduces the expression of VEGF and CD206 in bone marrow-derived M2 macrophages without inhibiting M1 macrophage function

**Figure 5:**
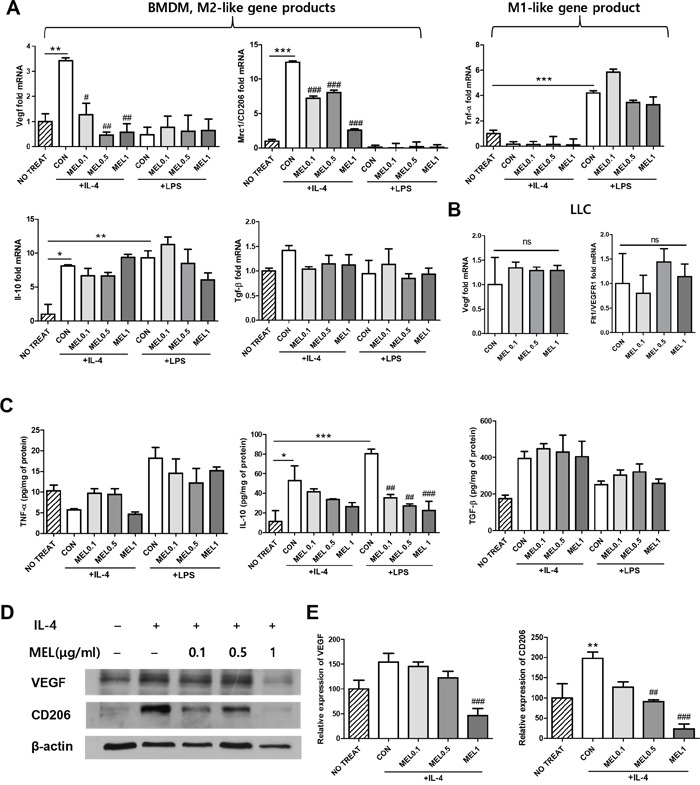
Regulation of CD206 and VEGF expression by melittin in M2-like macrophages *in vitro* **(A)** qPCR of M2 phenotypic markers (Vegf, Mrc1/CD206, Il-10, and Tgf-β) and a M1 phenotypic marker (Tnf-α) in bone marrow-derived macrophages (BMDM). The cells were stimulated with LPS or IL-4 for 24 h and incubated in the presence of PBS or melittin for another 24 h. Fold increase of each gene was normalized against the level of the unstimulated group (indexed as NO TREAT). **(B)** Relative mRNA levels of Vegf and flt1/VEGFR1 from melittin-treated tumor cells were compared with the PBS-treated control group and the results are shown as fold-difference. **(C)** TNF-α, IL-10, and TGF-β production was measured in the supernatant of the BMDM cultures using an ELISA. **(D-E)** VEGF and CD206 expressions were determined by Western blot analysis. β-actin was used as a loading control. Representative blots of 4-5 experiments are shown. Values are the mean and error bars represent SEM. *P < 0.05, **P < 0.01, and ***P < 0.001 represent a significant difference between the LPS or IL-4 treated CON group and the NO TREAT group. #P < 0.05, ##P < 0.01, and ###P < 0.001 represent a significant difference between the melittin treated group and the CON group. The non-marked data showed no significant (ns) differences when compared to the CON group. The data are presented as the mean ± SEM.

To assess whether melittin could alter macrophage M1/M2 activation, bone marrow-derived macrophages (BMDM) were stimulated with LPS or IL-4 and cultured with melittin or PBS. Total cell lysates and culture supernatants were analyzed for mRNA and protein expression. The gene/protein level of pro-inflammatory marker, TNF-α, was increased by LPS stimulation, but melittin treatment did not modify the expression. M2-related pro-angiogenic markers, VEGF, CD206, TGF-β, and IL-10 were increased after IL-4 stimulation. The gene/protein expression levels of TGF-β and IL-10 in M2 BMDMs were not altered by melittin treatment (Figure 5A and 5C). Notably, melittin treated-M2 BMDMs were expressed significantly reduced mRNA levels of both Vegf and Mrc1/CD206 compared with control (Figure 5A). Decreased levels of VEGF and CD206 protein expression by melittin were further confirmed by Western blot analysis (Figure 5D and 5E). Next, mRNA levels of Vegf and flt1/VEGFR1 in LLC cells were tested. There were no significant differences between control and melittin groups (Figure 5B). These results suggest that melittin has potential anti-angiogenesis activity by reducing M2 gene expression such as Mrc1/CD206 and Vegf, while not altering gene expression of Vegf and flt1/VEGFR1 in LLC cells.

**Figure 6:**
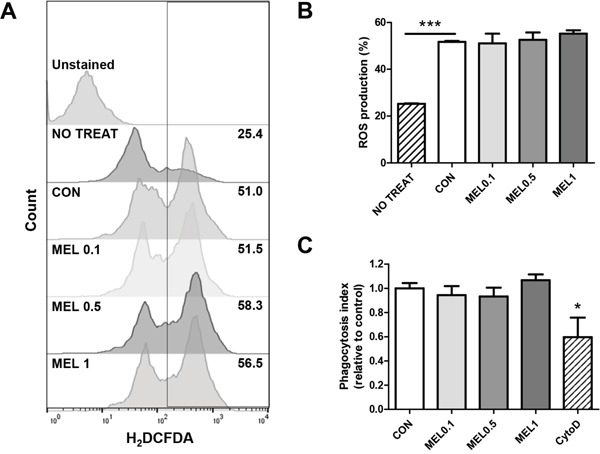
Effect of melittin on macrophage functions **(A-B)** Intracellular ROS productions of unstimulated cells (NO TREAT) or M1-polarized macrophages, treated with PBS (CON) or melittin (MEL) were measured by H_2_DCFDA staining respectively. **(C)** Relative phagocytosis index of melittin or cytochalashin D (cytoD)-treated BMDMs compared with control was estimated by measuring internalized latex-bead fluorescence intensity. The mean ± SEM for three replicates are shown. *P < 0.05, ***P < 0.0001.

For pro-inflammatory M1 macrophages, it is integral to have functional properties such as phagocytosis, endocytosis, cytokine secretion, and ROS production to help killing pathogens [33]. We confirmed the effect of melittin on macrophage function by measuring the level of ROS production and phagocytosis index of M1 BMDMs. The level of intracellular ROS did not differ between melittin-treated and control BMDMs (Figure 6A and 6B). The phagocytic capacity was unchanged by melittin treatment whereas the phagocytosis index was found to be significantly lower in actin polymerization inhibitor, cytochalasin D, treated group (Figure 6C). These data implicated that melittin treatment did not inhibit the function properties of macrophages such as ROS production and phagocytosis.

### Selective downregulation of CD206^+^ TAMs results in a reduction in tumor angiogenesis

**Figure 7:**
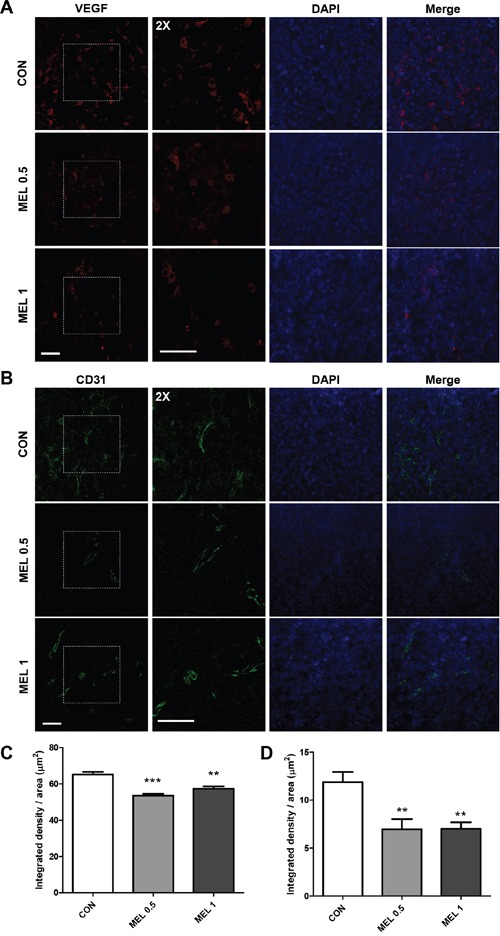
Effect of melittin treatment on the tumor angiogenic network **(A)** VEGF (red) and **(B)** CD31 (green) positive cells were visualized with immunofluorescence staining in a paraffin sectioned tumor. The nuclei were counterstained with DAPI (blue). **(C-D)** The intensity of VEGF **(C)** and CD31 **(D)** were quantified by image J. The data are presented as the mean ± SEM; **P < 0.01, *** P < 0.001, versus the corresponding control group. Total magnification, 400X. Scale bars, 50 μm.

TAMs promote tumor angiogenesis by secreting a variety of growth factors, proangiogenic factors and cytokines that stimulate angiogenesis and tumor growth. VEGF, which is regarded as the most potent angiogenic protein [34], not only induces angiogenesis by stimulating endothelial sprouting but also maintains the survival of new vessels in tumors [35]. CD31 (PECAM) is a marker for blood vessels and has been widely used to detect angiogenesis in tumor mice models [36]. Therefore, we used two major angiogenic markers, VEGF and CD31, to confirm whether the effect of melittin extends to inhibition of angiogenesis. Immunofluorescence staining revealed decreased levels of VEGF and CD31 in melittin treated tumor tissue compared to the PBS group (Figure 7A and 7B). Integrated density was measured with image J and there were significant differences between the PBS control group and both the 0.5 mg/kg and 1 mg/kg melittin treated group (Figure 7C and 7D). This suggests that reduction of the population of tissue resident M2-like TAMs results in an inhibition of angiogenesis.

## DISCUSSION

In this study, we demonstrated the effect of melittin on subcutaneously injected murine Lewis lung carcinoma. Treatment with melittin markedly decreased the number of CD45^+^CD11b^+^F4/80^+^ TAMs (Figure 3B), especially CD206^+^ M2-like TAMs in tumor stroma (Figure 4). This resulted in the reduction of VEGF^+^ and CD31^+^ cells in tumor tissues, suggesting that angiogenesis was hindered by the reduction of the TAM population. These findings demonstrate a novel effect of melittin: an anti-angiogenic effect in the early stage by regulation of M2-like macrophages in the tumor stroma. Due to the amphipathic and cytolytic properties of melittin, most cancer therapies with melittin focus on killing tumor cells by disrupting cell membranes or activating the apoptosis pathway [20, 21, 37]. Generally, cancer cells have a high membrane potential [38], so have a higher affinity with lytic peptides than normal cells. However, lytic peptides can cause off-target effects by disrupting the lipid membranes of normal cells [22–24]. Here, we focused on immune regulation of melttin without producing side effects *in vivo* and *in vitro*. We injected 0.5 mg/kg or 1 mg/kg of melittin, based on several studies which used safe dose of 0.1 to 5 mg/kg *in vivo* [39–41]. Pratt JP *et al*. suggested that lower than 2.0 μM of melittin does not cause membrane perturbing *in vitro* [42]. In our study, melittin did not cause cell lysis in the range of concentrations used *in vitro* and selectively reduced the M2 phenotypic markers of M2-like macrophages. Several studies have revealed the effect of melittin on macrophages. Srivastava *et al*. confirmed that melittin inhibits LPS binding on RAW-264.7 macrophages in an aqueous environment [28]. Park *et al*. suggested that melittin affected the LPS-induced expression of cyclooxygenase 2 (COX-2), cytosolic phospholipase A2 (PLA2), inducible NO synthase (iNOS), and levels of PGE_2_ and NO by preventing the LPS-induced transcriptional and DNA binding activity of NF-κB via the inhibition of IκB release and p50 translocation [26]. However, the effect is limited to LPS-induced macrophage activation and the effect of melittin on M2-like macrophages in the tumor microenvironment is not well known. Here, we confirmed the association between melittin treatment and M2-like macrophages and showed that melittin did not directly affect LLC cells or M1-like macrophages by quantifying the gene expression patterns and performing a cell cycle analysis. The quantification of levels of Tnf-α in M1 macrophages by real-time PCR revealed that melittin had no effect on M1-like macrophages but markedly reduced the level of M2-related genes in M2-like macrophages, such as Mrc1/CD206 and Vegf (Figure 5A). The levels of Vegf and flt1/VEGFR1 in LLC cells were unchanged by melittin treatment (Figure 5B). As we showed that melittin bound to CD11b^+^ macrophages with higher affinity than CD4^+^ or CD8^+^ T cells in Figure 3, these results suggest that melittin could bind specifically to macrophages. Additionally, preferential binding of melittin to CD206^+^ M2 phenotypic macrophages was observed in F4/80^+^ populations (Figure 3F). However, this event is not associated with phagocytic activity, since blocking actin polymerization of macrophage do not affect binding of melittin to macrophages. The detailed mechanisms of specific binding of melittin to M2 macrophages remain to be elucidated.

TAMs are considered promising targets for tumor therapy because TAMs facilitate tumor cell progression and angiogenesis and suppress the adaptive immune response [3, 4]. Clodronate liposomes, known to deplete macrophages, have been considered an effective anti-angiogenic treatment [13]. However, they failed to reduce the number of CD31^+^ vessels although the number of TAMs was significantly reduced [43]. In addition, they also eliminate resident macrophages that are critical to the host defense [44]. Although it is unclear whether melittin affects the tissue resident macrophages, it is meaningful that melittin only reduced the M2-like TAMs in the tumor tissue without affecting splenic M1 or M2 macrophages. CD31^+^ vessels also declined as a result of the lack of macrophages.

Taken together, these findings present new possibilities for melittin treatment in tumor therapy and regulation of macrophages. Although further studies are required to understand the detailed mechanisms of macrophage-melittin interactions, the data presented here indicates that melittin can be used as an antiangiogenic therapy, combined with other chemotherapies, and represents a promising new approach.

## MATERIALS AND METHODS

### Animals and cells

Wild-type C57BL/6 mice were purchased from SLC Japan Bred. Co. Ltd (Shizuoka, Japan). The animal procedures were approved by the University of Kyung Hee Institutional Animal Care and Usage Committee (KHUASP(SE)-15-067). All animals were maintained in a pathogen-free environment on a 12-h light/dark cycle and had access to food and water. The LLC, MLE12, A549, and H441 cells were cultured in medium (LLC, DMEM; MLE12, DMEM/F-12; A549 and H441, RPMI-1640; Welgene, Gyeongsan, Korea) supplemented with 10% heat-inactivated fetal bovine serum (FBS; Welgene), 100 U/ml penicillin and 100 μg/ml streptomycin (Invitrogen Life Technologies, Rockville, MD, USA). The cells were cultured every 2-3 days until the cells became 80% confluent. Cells were incubated at 37 °C with 95% humidity and 5% CO_2_ for all experiments.

### Tumor cell challenge

For generating tumor models, LLC cells were mixed with Matrigel matrix (Corning, NY, USA). Male C57BL/6 wild type mice (6-8wk-old) were inoculated subcutaneously in the right flank with 5×10^4^ cells per mouse. Recombinant melittin (GenScript Corporation, Piscataway, NJ, USA) was administered every 3 days (a total of 5 times) intraperitoneally, five days after tumor injection. Macrophage depleteion was performed by clodronate liposome (200 μl per mouse for first dose, 100 μl for maintenance dose every 4 days intraperitoneally) starting 3 days before tumor injection. Clodronate liposome and control liposome were purchased from FormuMax (Sunnyvale, CA, USA). Tumor size was monitored every 2-3 days by measuring two opposing diameters (volume = length × width × width/2). If the tumor size exceeded 5% of body weight, mice were sacrificed for the next set of experiments. Then, tumors and spleens were surgically removed.

### Blood cell profile test

Blood was collected from the retro-orbital plexus of mice while under anesthesia. Blood was mixed with EDTA immediately and analyzed with a Hemavet 950 auto-sampler (Drew scientific, Waterbury, CT, USA) following the manufacturer's manual. The parameters for leukocytes, erythrocytes, and thrombocytes were measured and expressed in percentages.

### Cell cycle analysis

Briefly, cells were plated on 6-well plates at a density of 5×10^5^/well and incubated with 0.1, 0.5, 1, 2 μg/ml of melittin or PBS for 24 h. Cells were collected and washed twice with PBS, fixed in 70% pre-chilled ethanol and stored overnight at -20 °C. The cells were washed and resuspended in 500 μl of PBS with 0.1% Triton X-100 and 20 μg/ml of RNase. Next, 50 μg/ml of propidium iodide (PI) was added. The stained cells were detected by a FACSCalibur flow cytometer (Becton Dickinson, San Jose, CA, USA) after being incubated at 37 °C for 20 min. Data were analyzed by FlowJo software (Treestar, Inc., San Carlos, CA, USA).

### Preparation of bone marrow-derived macrophages (BMDM)

To generate mouse BMDM, cells were harvested as previously described [45]. The cells were cultured for 7 days in RPMI-1640 complete culture medium with 10 ng/ml murine recombinant M-CSF (R&D systems, Minneapolis, MN, USA). After the cells differentiated to M0 macrophages, the cells were seeded in 6-well plates (1×10^6^ cells/well) and incubated overnight with 100 ng/ml LPS or 20 ng/ml murine recombinant IL-4 (R&D systems) to induce M1 or M2 phenotypic macrophage differentiation.

### Cell isolation and flow cytometry analysis

Tumors were minced into thin pieces and were dissociated in DNase I (1 U/ml; Roche, Indianapolis, IN, USA), collagenase D (1 mg/ml; Roche), and 0.125% trypsin-EDTA (Gibco) in pre-warmed DMEM (Welgene) for an hour at 37 °C with gentle agitation. The tissues were mechanically dissociated on a 100 μm nylon mesh strainer. Next, the single cells were passed through a 40 μm nylon mesh strainer. Spleens were also mechanically dissociated on 40 μm nylon mesh strainer. RBCs were lysed for 5 minutes in 1X Pharmlyse buffer.

Cells were stained with fluorescently tagged antibodies. All data were detected by a FACSCalibur cytometric system and analyzed by FlowJo software. The following fluorophore-labeled antibodies were purchased from e-bioscience (San Diego, CA, USA): CD45-FITC, CD3-FITC, CD4-PE, CD4-FITC, CD8-APC, CD11b-APC, CD11c-PE, CD25-PE, CD25-APC, B220-FITC, CD19-PE, CD86-APC. Foxp3-Alexa Fluor647 was purchased from BD bioscience, and F4/80-PE and CD206-APC were obtained from Biolegend (San Diego, CA, USA).

### Melittin binding study

Rhodamine-conjugated melittin peptides were purchased from GenScript (Piscataway, NJ, USA). Splenocytes were seeded in a 6-well culture plate with 0.5 μg/ml of rhodamine-conjugated melittin. An hour later, cells were harvested and unbound peptides were washed off twice. Cells were stained with APC-conjugated antibodies for an hour at 4 °C to confirm the binding of melittin to CD4^+^ and CD8^+^ T cells and CD11b^+^ monocytes.

To verify whether the binding of melittin to CD11b^+^ cells is related to phagocytosis, splenocytes were pretreated with 10 nM cytochalasin D or vehicle (DMSO) for an hour in a 37 °C incubator. Next, the cells were incubated with the rhodamine-conjugated peptides and stained with CD11b-APC antibody as described above.

To observe the binding of melittin on CD11b^+^ subsets in splenocytes, macrophage, dendritic cells, and neutrophils were probed with anti-mouse F4/80-FITC, CD11c-APCcy7, and Gr1-PEcy7 (e-bioscience). Annexin-V was added to the samples prior to data acquisition to discriminate dead cells. M1 of M2 macrophages were stained with CD86-PEcy7 (e-bioscience) or CD206-PercpCy5.5 (Biolegend). Cells were detected on a FACSCalibur or FACSCantoII.

### Tissue preparation and immunofluorescence confocal microscopy analysis

Tumors from inoculated mice were fixed overnight with paraformaldehyde, dehydrated and embedded in paraffin. Sections (5 μm thick) of the embedded tissue were cut on a rotary microtome and deparaffinized. Slides were antigen retrieved with an autoclave in trisodium citrate buffer (pH 6) for 1 min, washed with PBS, and then blocked for 1 h with 1.5% BSA containing 0.2% Triton X-100. Slides were incubated overnight at 4 °C with anti-VEGF and anti-CD31 primary antibodies (1:200, Santa Cruz Biotechnology, Santa Cruz, CA, USA). All tissue sections were visualized with alexa-488 or alexa-594 conjugated secondary antibody (1:500, Invitrogen) after incubation at RT for 1 h. All antibodies were diluted in 0.5% BSA solution and incubated in a humid chamber. Slides were mounted with DAPI solution and analyzed with laser scanning confocal microscopy (Bio-Rad, Richmond, CA, USA). All of the images were captured by LSM and the total integrated density was measured by image J software.

### Quantitative real-time PCR

Total RNA was extracted and reverse transcribed to cDNA from melittin or PBS-treated 1×10^6^ LLC cells or bone marrow-derived macrophages. Quantitative real-time PCR was performed according to a previous report [46]. Data were expressed as 2^-ΔΔCT^ for the experimental gene, normalized to GAPDH, and presented as fold change relative to LPS or IL-4 non-treated control. The following primers were used: Gapdh (for: ACCCAGAAGACTGTGGATGG; rev: CACATTGGGGGTAGGAACAC), Tnf-α (for: TTCTGTCTACTGAACTTCGGGGTGATCGGTCC; rev: GTATGAGATAGCAAATCGGCTGACGGTGTGGG), Mrc1/ CD206 (for: AGTGGCAGGTGGCTTATG; rev: GGTT CAGGAGTTGTTGTG), Il-10 (for: ATAACTGCAC CCACTTCCCA; rev: TCATTTCCGATAAGGCTTGG), Tgf-β (for: GAAGGCAGAGTTCAGGGTCTT; rev: GGTTCCTGTCTTTGTGGTGAA), Vegf (for: GGAGA TCCTTCGAGGAGCACTT; rev: GGCGATTTAGCAG CAGATATAAGAA [47], Flt1/VEGFR1 (for: ACATTGGTGGTGGCTGACTCTC; rev: CCTCTCCTT CGGCTGGCATC) [48].

### ELISA

Cytokine secretion in the culture medium was assayed using an ELISA kit according to the procedure recommended by the supplier. Murine IL-10, TNF-α was purchased from BD Biosciences, and TGF-β was purchased from R&D systems. Results were expressed in pg of cytokine normalized per mg of total proteins.

### Western blot analysis

Total protein was extracted from melittin or PBS treated-BMDM using RIPA buffer and quantified with Bio-Rad assay. 20 μg of protein were separated on 8% SDS Tris-Glycine gel and transferred onto nitrocellulose membranes (Invitrogen). The following antibodies and dilution factors were used: VEGF goat polyclonal antibody (sc-1836, 1: 1000, Santa Cruz), actin goat polyclonal antibody (sc-1616, 1:1000, Santa Cruz), rat anti-mouse CD206 (MCA2235GA, 1:200, AbD serotec), anti-goat IgG conjugated to HRP (SA007, 1:1000, GenDEPOT), and anti-rat IgG conjugated to HRP (405405, 1:1000, Biolegend). Protein bands were visualized using an ECL solution (GE healthcare) and measured by Image J software. The intensity of proteins was normalized over the intensity of actin.

### Intracellular reactive oxygen species (ROS) detection

BMDMs were plated 24-well plate and treated with melittin or PBS for 24h. Cells were loaded with 5μM C2’,7’-dichlorodihydrofluorescein diacetate (H_2_DCFDA; Molecular Probes) and incubated at 37°C for 30 min. H_2_DCFDA containing medium was removed and cells were washed twice with pre-warmed PBS. Cells were harvested, and the level of ROS production was immediately analyzed using flow cytometry.

### Phagocytosis assay

Cells were plated 96-black well plate and treated as described above. Cells were pre-treated with or without cytochalasin D for 30 min before treating latex beads-FITC (Sigma-Aldrich). After 2h incubation, external particle was removed by washing three times with PBS. The fluorescence of internalized bead was measured in the fluorescence plate reader (Fluorskan Ascent FL) using an excitation of 485nm and an emission of 527nm after quenching with trypan blue.

### Statistical analysis

The statistical significance was assessed by Student's *t*-test for single comparisons, or one-way ANOVA followed by Tukey's post-hoc test for multiple comparisons using Prism 5.01 software (GraphPad Software Inc., San Diego, CA, USA).

## References

[1] Colotta F, Allavena P, Sica A, Garlanda C, Mantovani A (2009). Cancer-related inflammation, the seventh hallmark of cancer: links to genetic instability. Carcinogenesis.

[2] Jeong SK, Yang K, Park YS, Choi YJ, Oh SJ, Lee CW, Lee KY, Jeong MH, Jo WS (2014). Interferon gamma induced by resveratrol analog, HS-1793, reverses the properties of tumor associated macrophages. International immunopharmacology.

[3] Huang S, Van Arsdall M, Tedjarati S, McCarty M, Wu W, Langley R, Fidler IJ (2002). Contributions of stromal metalloproteinase-9 to angiogenesis and growth of human ovarian carcinoma in mice. J Natl Cancer Inst.

[4] Lin EY, Li JF, Gnatovskiy L, Deng Y, Zhu L, Grzesik DA, Qian H, Xue XN, Pollard JW (2006). Macrophages regulate the angiogenic switch in a mouse model of breast cancer. Cancer Res.

[5] Folkman J (2003). Fundamental concepts of the angiogenic process. Current molecular medicine.

[6] Gordon S (2003). Alternative activation of macrophages. Nat Rev Immunol.

[7] Mosser DM, Edwards JP (2008). Exploring the full spectrum of macrophage activation. Nat Rev Immunol.

[8] Lamagna C, Aurrand-Lions M, Imhof BA (2006). Dual role of macrophages in tumor growth and angiogenesis. Journal of leukocyte biology.

[9] Roszer T (2015). Understanding the Mysterious M2 Macrophage through Activation Markers and Effector Mechanisms. Mediators of inflammation.

[10] Sica A, Saccani A, Mantovani A (2002). Tumor-associated macrophages: a molecular perspective. Int Immunopharmacol.

[11] Mantovani A, Bottazzi B, Colotta F, Sozzani S, Ruco L (1992). The origin and function of tumor-associated macrophages. Immunol Today.

[12] Qian BZ, Pollard JW (2010). Macrophage diversity enhances tumor progression and metastasis. Cell.

[13] Zeisberger SM, Odermatt B, Marty C, Zehnder-Fjallman AH, Ballmer-Hofer K, Schwendener RA (2006). Clodronate-liposome-mediated depletion of tumour-associated macrophages: a new and highly effective antiangiogenic therapy approach. Br J Cancer.

[14] Mitchem JB, Brennan DJ, Knolhoff BL, Belt BA, Zhu Y, Sanford DE, Belaygorod L, Carpenter D, Collins L, Piwnica-Worms D, Hewitt S, Udupi GM, Gallagher WM (2013). Targeting tumor-infiltrating macrophages decreases tumor-initiating cells, relieves immunosuppression, and improves chemotherapeutic responses. Cancer Res.

[15] Eisenberg D (1984). Three-dimensional structure of membrane and surface proteins. Annu Rev Biochem.

[16] Dempsey CE (1990). The actions of melittin on membranes. Biochim Biophys Acta.

[17] Katsu T, Kuroko M, Morikawa T, Sanchika K, Fujita Y, Yamamura H, Uda M (1989). Mechanism of membrane damage induced by the amphipathic peptides gramicidin S and melittin. Biochim Biophys Acta.

[18] Ladokhin AS, Selsted ME, White SH (1997). Sizing membrane pores in lipid vesicles by leakage of co-encapsulated markers: pore formation by melittin. Biophys J.

[19] Jo M, Park MH, Kollipara PS, An BJ, Song HS, Han SB, Kim JH, Song MJ, Hong JT (2012). Anti-cancer effect of bee venom toxin and melittin in ovarian cancer cells through induction of death receptors and inhibition of JAK2/STAT3 pathway. Toxicol Appl Pharmacol.

[20] Russell PJ, Hewish D, Carter T, Sterling-Levis K, Ow K, Hattarki M, Doughty L, Guthrie R, Shapira D, Molloy PL, Werkmeister JA, Kortt AA (2004). Cytotoxic properties of immunoconjugates containing melittin-like peptide 101 against prostate cancer: *in vitro* and *in vivo* studies. Cancer Immunol Immunother.

[21] Soman NR, Baldwin SL, Hu G, Marsh JN, Lanza GM, Heuser JE, Arbeit JM, Wickline SA, Schlesinger PH (2009). Molecularly targeted nanocarriers deliver the cytolytic peptide melittin specifically to tumor cells in mice, reducing tumor growth. J Clin Invest.

[22] Lee MT, Hung WC, Chen FY, Huang HW (2008). Mechanism and kinetics of pore formation in membranes by water-soluble amphipathic peptides. Proc Natl Acad Sci U S A.

[23] Kokot G, Mally M, Svetina S (2012). The dynamics of melittin-induced membrane permeability. Eur Biophys J.

[24] Hoskin DW, Ramamoorthy A (2008). Studies on anticancer activities of antimicrobial peptides. Biochim Biophys Acta.

[25] van den Bogaart G, Guzman JV, Mika JT, Poolman B (2008). On the mechanism of pore formation by melittin. J Biol Chem.

[26] Park HJ, Lee SH, Son DJ, Oh KW, Kim KH, Song HS, Kim GJ, Oh GT, Yoon DY, Hong JT (2004). Antiarthritic effect of bee venom: inhibition of inflammation mediator generation by suppression of NF-kappaB through interaction with the p50 subunit. Arthritis Rheum.

[27] Bhunia A, Domadia PN, Bhattacharjya S (2007). Structural and thermodynamic analyses of the interaction between melittin and lipopolysaccharide. Biochim Biophys Acta.

[28] Srivastava RM, Srivastava S, Singh M, Bajpai VK, Ghosh JK (2012). Consequences of alteration in leucine zipper sequence of melittin in its neutralization of lipopolysaccharide-induced proinflammatory response in macrophage cells and interaction with lipopolysaccharide. J Biol Chem.

[29] Kim WH, Park JY, Ok SH, Shin IW, Sohn JT (2015). Association Between the Neutrophil/Lymphocyte Ratio and Acute Kidney Injury After Cardiovascular Surgery: A Retrospective Observational Study. Medicine (Baltimore).

[30] Zhang M, He Y, Sun X, Li Q, Wang W, Zhao A, Di W (2014). A high M1/M2 ratio of tumor-associated macrophages is associated with extended survival in ovarian cancer patients. J Ovarian Res.

[31] Ma J, Liu L, Che G, Yu N, Dai F, You Z (2010). The M1 form of tumor-associated macrophages in non-small cell lung cancer is positively associated with survival time. BMC Cancer.

[32] Yuan A, Hsiao YJ, Chen HY, Chen HW, Ho CC, Chen YY, Liu YC, Hong TH, Yu SL, Chen JJ, Yang PC (2015). Opposite Effects of M1 and M2 Macrophage Subtypes on Lung Cancer Progression. Sci Rep.

[33] Martinez FO, Gordon S (2014). The M1 and M2 paradigm of macrophage activation: time for reassessment. F1000Prime Rep.

[34] Torimura T, Sata M, Ueno T, Kin M, Tsuji R, Suzaku K, Hashimoto O, Sugawara H, Tanikawa K (1998). Increased expression of vascular endothelial growth factor is associated with tumor progression in hepatocellular carcinoma. Hum Pathol.

[35] Benjamin LE, Keshet E (1997). Conditional switching of vascular endothelial growth factor (VEGF) expression in tumors: induction of endothelial cell shedding and regression of hemangioblastoma-like vessels by VEGF withdrawal. Proc Natl Acad Sci U S A.

[36] Fulzele SV, Chatterjee A, Shaik MS, Jackson T, Singh M (2006). Inhalation delivery and anti-tumor activity of celecoxib in human orthotopic non-small cell lung cancer xenograft model. Pharm Res.

[37] Li B, Gu W, Zhang C, Huang XQ, Han KQ, Ling CQ (2006). Growth arrest and apoptosis of the human hepatocellular carcinoma cell line BEL-7402 induced by melittin. Onkologie.

[38] Yang M, Brackenbury WJ (2013). Membrane potential and cancer progression. Front Physiol.

[39] Park JH, Kum YS, Lee TI, Kim SJ, Lee WR, Kim BI, Kim HS, Kim KH, Park KK (2011). Melittin attenuates liver injury in thioacetamide-treated mice through modulating inflammation and fibrogenesis. Exp Biol Med (Maywood).

[40] Choi JH, Jang AY, Lin S, Lim S, Kim D, Park K, Han SM, Yeo JH, Seo HS (2015). Melittin, a honeybee venomderived antimicrobial peptide, may target methicillinresistant Staphylococcus aureus. Mol Med Rep.

[41] Huh JE, Kang JW, Nam D, Baek YH, Choi DY, Park DS, Lee JD (2012). Melittin suppresses VEGF-A-induced tumor growth by blocking VEGFR-2 and the COX-2-mediated MAPK signaling pathway. J Nat Prod.

[42] Pratt JP, Ravnic DJ, Huss HT, Jiang X, Orozco BS, Mentzer SJ (2005). Melittin-induced membrane permeability: a nonosmotic mechanism of cell death. *In Vitro* Cell Dev Biol Anim.

[43] Germano G, Frapolli R, Belgiovine C, Anselmo A, Pesce S, Liguori M, Erba E, Uboldi S, Zucchetti M, Pasqualini F, Nebuloni M, van Rooijen N, Mortarini R (2013). Role of macrophage targeting in the antitumor activity of trabectedin. Cancer Cell.

[44] Cailhier JF, Partolina M, Vuthoori S, Wu S, Ko K, Watson S, Savill J, Hughes J, Lang RA (2005). Conditional macrophage ablation demonstrates that resident macrophages initiate acute peritoneal inflammation. J Immunol.

[45] Chung ES, Lee G, Lee C, Ye M, Chung HS, Kim H, Bae SJ, Hwang DS, Bae H (2015). Bee Venom Phospholipase A2, a Novel Foxp3+ Regulatory T Cell Inducer, Protects Dopaminergic Neurons by Modulating Neuroinflammatory Responses in a Mouse Model of Parkinson's Disease. Journal of immunology.

[46] Ye M, Chung HS, Lee C, Hyun Song J, Shim I, Kim YS, Bae H (2016). Bee venom phospholipase A2 ameliorates motor dysfunction and modulates microglia activation in Parkinson's disease alpha-synuclein transgenic mice. Experimental & molecular medicine.

[47] Shih SC, Robinson GS, Perruzzi CA, Calvo A, Desai K, Green JE, Ali IU, Smith LE, Senger DR (2002). Molecular profiling of angiogenesis markers. Am J Pathol.

[48] Ambade A, Satishchandran A, Szabo G (2016). Alcoholic hepatitis accelerates early hepatobiliary cancer by increasing stemness and miR-122-mediated HIF-1alpha activation. Sci Rep.

